# The effect of stone size on the results of extracorporeal shockwave lithotripsy versus semi-rigid ureteroscopic lithotripsy in the management of upper ureteric stones

**DOI:** 10.1080/2090598X.2021.1996820

**Published:** 2021-11-26

**Authors:** Ahmed S. El-Abd, Ahmed M. Tawfeek, Shawky A. El-Abd, Tarik A. Gameel, Hasan H. El-Tatawy, Magdy A. El-Sabaa, Mohamed G. Soliman

**Affiliations:** Urology Department, Faculty of Medicine, Tanta University, Tanta, Egypt

**Keywords:** Stones, extracorporeal shockwave lithotripsy, ureteroscopy

## Abstract

**Objectives:**

To evaluate the role of stone size on the efficacy and safety of extracorporeal shockwave lithotripsy (ESWL) monotherapy vs ureteroscopy (URS) for managing upper ureteric stones.

**Patients and methods:**

The study design was a randomised prospective study of a total cohort of 180 patients with upper ureteric single stones of 0.5–1.5 cm. Half of the patients were managed by ESWL monotherapy, while the other half underwent URS with stone fragmentation using an ultrasound lithotripter (URSL). The success rate, re-treatment rate, auxiliary procedure (AP) rate, efficacy quotient, and complications were compared between the two groups.

**Results:**

After single URSL and ESWL procedures 70/90 (77.8%) and 35/90 (38.9%) of the stones were successfully cleared, respectively (*P* < 0.001). The re-treatment rate after ESWL was significantly higher than in the URSL group (38.9% vs 11.1%, *P* < 0.001). Requiring an AP was not significantly different following ESWL (22.2%) and URSL (24.4%) treatment. The overall stone-free rate (SFR) at 3 months was significantly superior in the URSL group (88.9% vs 77.8%); however, both procedures had excellent results with no significant difference for stones of <1 cm (95.5% vs 92.9%, *P* > 0.05), compared to better results following URSL for stones of >1 cm (82.6% vs 64.6%, *P* < 0.05).

**Conclusion:**

Our present study supports that ESWL is recommended as a first-line non-invasive monotherapy for upper ureteric opaque stones of <1 cm, while URSL is recommended as a first-line treatment for stones of >1 cm. The results for URSL were superior with lower a re-treatment rate, rapid stone clearance in a very short time, and less radiation exposure. Therefore, stone size is an important factor for the final decision of the initial management of upper ureteric stones because it has a direct relation to the efficacy of ESWL, but it has no effect on the results of URSL.

## Introduction

Upper ureteric stones are usually associated with obstructive uropathy with gradual and progressive impairment in renal function. Patients presenting with upper ureteric stones receive debated treatment modalities, starting from extracorporeal shockwave lithotripsy (ESWL) to contact lithotripsy via an antegrade or retrograde approach [1]. ESWL is an outpatient non-invasive treatment that does not require anaesthesia, but it may not render the patient stone-free in one session [2].

With the use of a small calibre long ureteroscope, recent auxiliary instruments, and disintegration tools; treatment of upper ureteric stones can be effectively achieved with improved stone-free rates (SFRs) and minimal complications [3]. The incidence of stone migration was reported to be as high as 48% after using pneumatic lithotripsy with subsequent mandatory additional procedures, which increase morbidity and cost burden [4].

The aim of the present randomised study was to compare ESWL vs ureteroscopic lithotripsy (URSL) using ultrasonic disintegration in the treatment of uncomplicated upper ureteric stones of 0.5–1.5 cm with the effect of stone size on the results.

## Patients and methods

From December 2015 to June 2020, patients with single, proximal radio-opaque ureteric stones of up to 1000 Hounsfield units (HU) between the PUJ and the sacro-iliac joint with a stone size of 0.5–1.5 cm in the longest diameter were included in the study. Patients with active urinary infection, morbid obesity, multiple or impacted stones of >1.5 cm, prior JJ stents, uncorrected coagulopathy, impaired renal function, pregnancy, and cases presenting with anuria or bilateral stones were all excluded.

After obtaining approval of the local Ethics Committee, the nature of the trial was explained to the patients and informed consent was obtained from the willing participants. The patients were then randomly assigned to two treatment groups: Group 1, ESWL and Group 2, URSL. Patients underwent either of the procedures as a primary treatment without any biased selection by using a random numbers table, with 90 patients treated in each group.

All patients were assessed preoperatively with full blood count, renal and liver function, coagulation profile and urine analysis, culture, and sensitivity. Radiological evaluation included plain abdominal radiograph of the kidneys, ureters, and bladder (KUB), and spiral non-contrast CT (NCCT).

The ESWL was performed using a Dornier Compact Delta II (Dornier MedTech, Munich, Germany). Patients were treated in a supine position under intravenous analgesia using nalbuphine 20-mg infusion as an outpatient procedure. The maximum number of shocks per session was 4000, at rate of 80–100/min, or until complete disintegration of the stone was observed by fluoroscopy. The shockwave energy was gradually increased, according to patient tolerance as analgesia was administered routinely to have a stable patient during the session. The number of sessions needed for stone fragmentation and number of shocks used in every session were recorded.

The URSL was done under spinal or general anaesthesia using 8.5-F semi-rigid ureteroscope (Richard Wolf, Knittlingen, Germany), and disintegration was done under direct vision. Dilatation of ureteric orifice when needed was done using a balloon catheter or double lumen ureteric dilator. Intracorporeal lithotripsy was done using an ultrasound lithotripter, using a stone cone as an ante-retropulsion tool. All fragments were extracted using URS forceps and the cone was released inside the urinary bladder. After completion of the procedure guided by both direct vision and fluoroscopic control, a 6-F ureteric catheter was placed for 2 days. If extensive manipulation or mucosal injury had occurred, or incomplete disintegration and in cases with solitary renal unit, a 6-F JJ was placed for 4 weeks.

Success was defined as clearance of all fragments guided by KUB and NCCT 4 weeks after the procedure. The indication for re-treatment was planned when the original treatment was insufficient to render the patient stone free (when the residual stone was >0.4 cm) whether using ESWL or URSL.

An auxiliary procedure (AP) was considered when a different procedure was implemented to clear all the residual stones or to treat any complication.

The primary outcome of the study was to evaluate the overall SFR. Secondary outcomes included re-treatment rate, APs, and estimated efficacy quotient (EQ).

Other evaluated parameters included immediate SFRs (ISFRs), operative and fluoroscopy time. Results were compared according to the size of stones in each group. Complications reported in both groups were compared based on the Clavien–Dindo classification.

The EQ for both groups was calculated using the following formula:

$$EQ=\frac{\;\left(percentage\;\;of\;SFR\;\;\times \;\;100\right)}{\left(100\;+percentage\;of\;retreatment+percentage\;of\;AP\right)}$$

Sample size calculation: the sample size was prospectively evaluated using the goodness-of-fit test for contingency tables with ‘effect size w’ of 0.5, α error protection of 0.05 and power of 0.80. Based on these data a total sample size of 61 patients was needed to be included in each of the study groups.

### Statistical analysis

Data were statistically analysed using the IBM Statistical Package for the Social Sciences (SPSS®) version 25 (IBM Corp., Armonk, NY, USA). The chi-square test and unpaired *t*-test were used to assess differences among groups for categorical variables and continuous variables, respectively. The differences were considered statistically significant for *P*< 0.05.

## Results

**Figure 1.**
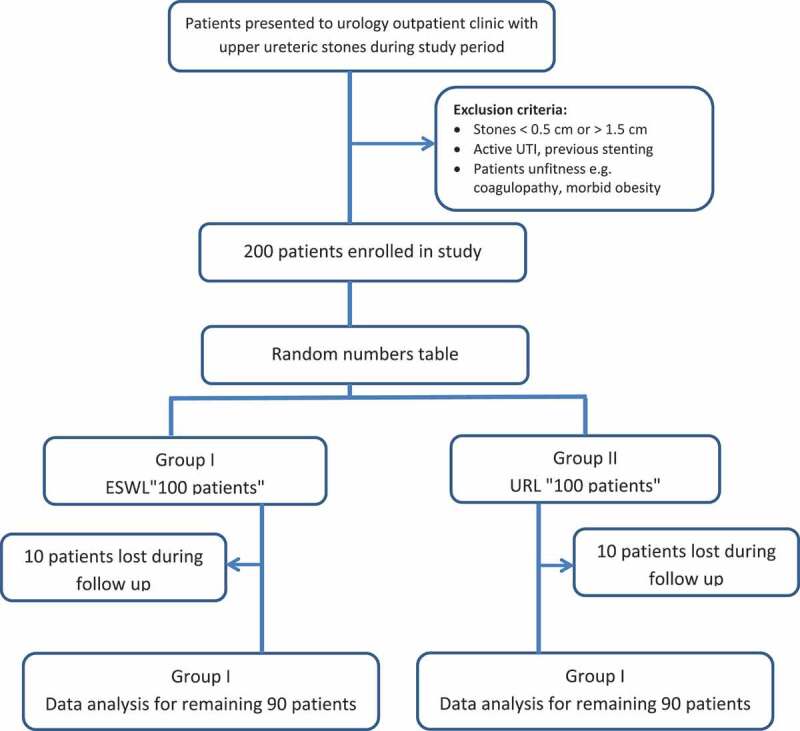
Patients flow through the study.

A total of 180 out of 200 patients with proximal ureteric stones completed the study protocol, 90 patients in each group. The rest of these patients did not complete the follow-up after first treatment (Figure 1). In both groups, no statistically significant difference was observed in patient data recorded in (Table 1).

**Table 1.** Patients’ characteristics in both groups

Characteristic	ESWL	URSL	*P*
Number of patients	90	90	
Age, years, mean (SD)	42 (12)	44.7 (10)	0.106
Sex, male/female, *n*	51/39	49/41	0.764
Side, right/left, *n*	41/49	42/48	0.764
Proximal dilatation, *n*	60	56	0.881
Stone size, mm, mean (SD)	11.1 (2.09)	11.3 (2.13)	0.529
Stone density, HU, mean (SD)	796.27 (101.48)	819.02 (116.12)	0.082

In the ESWL group, the ISFR was 38.9% with 35/90 patients cleared from stone after only one session. The overall SFR included a total of 70 patients (77.8%) who became stone free after three sessions, while another eight patients (8.9%) had clinically insignificant residual fragments (CIRF) of <0.4 cm. These patients were asymptomatic at the 3-month follow-up without any increase in size. Two-thirds of the failed cases (nine of 12) and all cases with CIRF (eight) were seen in patients with stones of >1 cm.

There was no impact of age, sex, and side of the stone on the results, but complete clearance with no CIRF was significantly higher in 39/42 cases with a stone size of <1 cm (92.9%), compared to larger stones of >1 cm in 31/48 patients (64.6%, *P*< 0.01; Table 2)

**Table 2.** Data and results after URSLs and ESWL in relation to stone size

	ISFR, 1 month	Overall SFR, 3 months	Re-treatment, *n*	AP, *n*	EQ
**ESWL, *n/N* (%)**					
<1 cm (*n* = 42)	23 (54.8)	39 (92.9)	16 ESWL		0.80
>1 cm (*n* = 48)	12 (25)	31 (64.6)	19 ESWL	10 URSL + 2 open + 8 JJ	0.46
Total (*n* = 90)	35/90 (38.9)	70/90 (77.8)	35 ESWL	10 URSL + 2 open + 8 JJ	0.48
**URSL, *n/N* (%)**					
<1 cm (*n* = 44)	38 (86.4)	42 (95.5)	4 URSL	1 ESWL + 1 open +4 JJ	0.79
>1 cm (*n* = 46)	32 (69.6)	38 (82.6)	6 URSL	5 ESWL + 3 open + 8 JJ.	0.57
Total (*n* = 90)	70/90 (77.8)	80/90 (88.9)	10 URSL	6 ESWL + 4 open + 12 JJ	0.59
**Statistics ESWL vs URSL**	Chi-square	*P*	Chi-square	*P*	
<1 cm (*n* = 86)	8.931	0.001	0.003	0.479	
> 1 cm (*n* = 94)	16.96	<0.001	3.041	0.041	
Total (*n* = 180)	26.423	<0.001	3.24	0.036	

The mean number of ESWL sessions per patient for smaller stones (<1 cm) was 1.5 compared to 1.9 for cases with stones of >1 cm (*P*< 0.05). Also significantly more shocks per session were needed for larger stones (*P*< 0.01).

All patients submitted to URSL were discharged the next morning, with the mean (range) operative time of 50 (25–115) min and dilatation of ureteric orifice was performed in 25/87 cases (28.7%) using a double-lumen ureteric catheter (*n*= 15) or balloon catheter (*n*= 10). The procedure was converted to open surgery in four cases due to large extravasation in two patients, and a hard stone and distal ureteric kink each in one patient. Only one of these patients had a small stone of <1 cm.

In this group, the ISFR was reported to be 77.8%. For stones of <1 cm, the ISFR was 86.4% (38/44 patients) vs 69.6% (32/46 patients) for stones of >1 cm. The overall SFR was 88.9% (80/90) and it was 95.5% (42/44) for stones of <1 cm vs 82.6% (38/46) for stones of >1 cm (Table 2).

Comparing the results in the two groups for the overall SFR as the primary outcome, there was no significant difference for stones of <1 cm (*P*= 0.5). Conversely, for stones of >1 cm, URSL showed a significantly better success rate in comparison to the ESWL group (*P*= 0.04).

Regarding re-treatment and APs for the management of failed primary procedures, in the ESWL group re-treatment was needed in 38.9% (35/90), while APs were used in 22.2% (20/90) including eight patients managed with JJ insertion. The remaining 12 patients (13.3%) who failed ESWL treatment were cleared from stones after URSL in 10 cases and open uretero-lithotomy in two (refused re-treatment with URSL).

In the URSL group, the re-treatment rate, and the need for an AP was reported in 10/90 patients (11.5%) and 22/90 patients (24.4%), respectively. All patients submitted to a second session of URSL became stone free (in four patients with stones <1 cm and in six with stones >1 cm; Table 2).

An AP was needed in 22 patients (six patients managed with ESWL, 12 with JJ insertion and four were converted to open uretero-lithotomy). The re-treatment rate was significantly higher in the ESWL group compared with the URSL group (*P*< 0.001); however, there was no significant difference in the APs (*P*= 0.4)

In the ESWL group, the estimated EQ was 0.8 and 0.46 for stones of < and >1 cm, respectively. While in the URSL group, the EQ was 0.79 and 0.57 for stones of < and >1 cm, respectively.

The mean (SD) fluoroscopy time after ESWL was 56 (3.4) s, compared to significantly less radiation exposure during URSL, with a mean (SD) fluoroscopy time of 18 (6.6) s (*P*< 0.001, Table 3).

**Table 3.** Statistical analysis of the results

	ESWL (*N* = 90)	URSL (*N* = 90)	Statistic	*P*
Overall success rate, *n/N* (%)	70/90 (77.8)	80/90 (88.9)	Chi-square = 3.24	0.033
<1 cm, *n/N* (%)	39/42 (92.9)	42/44 (95.5)	Chi-square = 0.003	0.479
>1 cm, *n/N* (%)	31/48 (64.6)	38/46 (82.6)	Chi-square = 3.041	0.041
Re-treatment, *n/N* (%)	35/90 (38.9)	10/90 (11.1)	Chi-square = 17.07	<0.001
AP, *n/N* (%)	20/90 (22.2)	22/90 (24.4)	Chi-square = 0.031	0.430
EQ	0.48	0.59		
Open, *n/N* (%)	2/90 (2.2)	4/90 (4.4)	Chi-square = 0.172	0.339
Fluoroscopy time, s, mean (SD)	56 (3.4)	18 (6.6)	*t* = 48.385	<0.001
Re-hospitalisation, *n* (%)	4 (4.5)	1 (1.1)	Chi-square = 1.75	0.385

All complications in our series were considered as minor (Clavien–Dindo Grade I–II) in both the ESWL and URSL groups.

In the ESWL group, 16 patients (17.8%) developed complications with more than one complication in some patients. Clinical haematuria after 24 h was encountered in 12 cases but variable degrees of renal colic, dysuria and vomiting were seen in 27 (30%), 10 (11%) and four patients (4.5%), respectively; all managed conservatively including α-blockers in two cases (2.2%) with steinstrasse. Hospital admission was necessary in four patients due to severe renal colic.

Complications in the URSL group included dysuria and renal colic, all were managed conservatively, but one patient needed hospitalisation for intravenous fluids and analgesics.

## Discussion

Various treatment modalities have been reported for management of upper ureteric stones. The decision of which treatment to implement depends on many factors, e.g. stone size, degree of the proximal backpressure, presence of distal obstruction, the available technology, and surgical experience. All of these are important for selecting the most suitable technique for the best SFR and minimal morbidity. The treatment options vary from direct contact lithotripsy to *in situ* non-contact ESWL for medium size stones, up to laparoscopic or open uretero-lithotomy for complicated cases with large stones [5]. In recent years, new generations of ESWL machines are associated with minimal tissue damage, less anaesthesia, and higher re-treatment rate [6]. However, as a non-invasive treatment it can be done as an outpatient procedure with high patient tolerance even on re-treatment, and no need for theatres or prior stenting and stent removal. This is reflected on the overall costs and time for stone clearance [7]. ESWL has a high success rate of 85–96% for small proximal ureteric stones after prior JJ stenting, but this success rate is lower for larger stones [8]. We performed a prospective randomised study of ESWL vs URSL of 0.5–1.5 cm stones in the upper ureter using ultrasonic disintegration without prior stenting.

In our present study, the ISFR of ESWL for stones < and >1 cm was 54.8% and 25%, respectively, although the overall stone clearance rate at 3 months was 92.9% and 64.6%, respectively. The total stone clearance rate was 77.8% with significant better results in smaller stones at this site. We excluded patients with prior ureteric surgery or morbid obesity and advanced hydronephrosis not only because of the negative impact on stone disintegration and subsequent stone clearance rate, but also to include in the study only patients that could be treated with either ESWL or URSL. These situations have previously been associated with higher re-treatment rates and prolonged clearance [9].

The ESWL was performed without anaesthesia and the patient left the ESWL unit immediately after disintegration, but the re-treatment rate was significantly greater compared to URSL (*P* < 0.001). The need for an AP was not significantly different between the ESWL (22.2%) and URSL (24.4%) groups; however, those with larger stones in the ESWL group required re-treatment with more ESWL sessions (1.9 vs 1.5) and more shocks per session. This was observed in our present study and many other previous reports [9–11]. Therefore, we found that the stone size is an important predictor for the SFR after ESWL, with reduced efficiency in large stones. In the situation of large upper ureteric stones, the stone is also surrounded by small disintegration chamber compared to similar size stones in the pelvicalyceal system, which is reflected by the better stone disintegration and clearance [12].

In recent years, the refinement of ureteroscopes (diameter and vision) and internal disintegration tools, including laser, have made treatment of stones in the upper ureter a viable competitor to ESWL. The debate depends on the non-invasiveness of ESWL and the availability of semi-rigid ureteroscopes in most urology centres, which are more durable, less expensive, can be done as an outpatient procedure with higher success rates and superior stone clearance in a shorter time [13]. Also many patients prefer to be free from the stone in one session with no need for re-treatment or APs [14]. Although the highest success after URSL was seen for lower ureteric stones, the new tools with the semi-rigid and flexible ureteroscopes has been associated with high success rates for upper ureteric stones [15].

Size by size, the overall SFR after URSL (95.5%) and ESWL (92.9%) was excellent in small stones, but URSL was significantly superior for larger stones of >1 cm, at 82.6% vs 64.6%, (*P* < 0.05). The re-treatment rate after URSL was 11.5%, which was significantly lower than the 38.9% in the ESWL group; however, the need for an AP was not significantly different between the groups, especially in patients with large stones. The estimated EQ in small stones was 0.79 and 0.8 for URSL and ESWL with no significant difference; however, URSL displayed a better efficacy (0.57) than ESWL (0.46) in large size stones due to a higher re-treatment rate that lowered the EQ of patients treated with ESWL. Therefore the stone size did not affect the efficacy of URSL, although a better EQ of ESWL was seen with small stones (0.8) than the large stones (0.46). The results of our present study are consistent with many other previous studies [16,17]. Other advantages of URSL are that it can be done as an outpatient procedure under minimal anaesthesia with a high success rate and minimal need for re-treatment and secondary procedures, which coincides with the desire of most patients in our present series and other previous reports [18]. Also many studies supported URSL as a safe procedure with stenting unnecessary and absence of intraoperative complications [19,20]. Furthermore, it can be used for failed cases after ESWL, radiolucent stones and in pregnancy [21].

However, the smaller semi-rigid ureteroscopes with no need for dilatation permits rapid access to the stone with a higher success rate due to intracorporeal disintegration under direct vision with the use of an adjunctive measures to prevent proximal migration and retrieval of small fragments [22].

Limitations of the present our study include the absence of the use of flexible URSL with laser disintegration and the lack of a paediatric age group; however, these parameters are currently under evaluation for comparison.

Complications with renal colic and dysuria that need re-hospitalisation were significantly higher in the ESWL group with large stones (4:1). All other complications were managed conservatively without major morbidity. The need for open intervention was not significantly different between the groups (2.2% and 4.4%). On the other hand, the mean fluoroscopy time and radiation exposure was significantly shorter in the URSL group than in the ESWL group (*P*< 0.001).

## Conclusion

Our present study supports that ESWL is recommended as a first-line non-invasive monotherapy for upper ureteric opaque stones of <1 cm, while URSL is recommended as a first-line treatment for stones of >1 cm. The results for URSL were superior with a lower re-treatment rate, rapid stone clearance in a very short time, and less radiation exposure. Therefore, stone size is an important factor for the final decision of the initial management of upper ureteric stones because it has a direct relation to efficacy of ESWL, but it has no effect on results of URSL.

## Abbreviations

AP: auxiliary procedure; CIRF: clinically insignificant residual fragments; EQ: efficacy quotient; ESWL: extracorporeal shockwave lithotripsy; HU: Hounsfield units; KUB, plain abdominal radiograph of the kidneys, ureters, and bladder; NCCT, non-contrast CT; (I)SFR: (immediate) stone-free rate; URS(L): ureteroscopy (lithotripsy)
